# Oxytocinergic cells of the posterior hypothalamic paraventricular nucleus participate in the food entrained clock

**DOI:** 10.1038/s41598-021-99266-0

**Published:** 2021-10-07

**Authors:** Mario Caba, Enrique Meza, Carolina Escobar, Angeles Jiménez, Mario Daniel Caba-Flores, María Luisa Moreno-Cortés, Angel I. Melo

**Affiliations:** 1 Centro de Investigaciones Biomédicas, Universidad Veracruzana, Xalapa, Ver. Mexico; 2 Facultad de Medicina, Universidad Nacional Autónoma de México, CDMX, Mexico; 3 Centro de Investigación en Reproducción Animal, CINVESTAV-UAT, Tlaxcala, Tlax Mexico; 4 Doctorado en Ciencias Biomédicas, CIB, Universidad Veracruzana, Xalapa, Ver. Mexico; 5 Instituto de Investigaciones Biológicas, Universidad Veracruzana, Xalapa, Ver. Mexico

**Keywords:** Neuroscience, Circadian rhythms and sleep, Feeding behaviour

## Abstract

The mechanisms underlying food anticipatory activity are still poorly understood. Here we explored the role of oxytocin (OT) and the protein c-Fos in the supraoptic nucleus (SON), medial (PVNm) and posterior (PVNp) regions of the paraventricular hypothalamic nucleus. Adult rats were assigned to one of four groups: scheduled restricted feeding (RF), ad libitum (AL), fasting after restricted feeding (RF-F), to explore the possible persistence of oscillations, or ad libitum fasted (AL-F). In the SON and in the PVNm, OT cells were c-Fos positive after food intake; in contrast, OT cells in the PVNp showed c-Fos activation *in anticipation to* food access, which persisted in RF-F subjects. We conclude that OT and non-OT cells of the SON and PVNm may play a role as recipients of the entraining signal provided by food intake, whereas those of the PVNp which contain motor preautonomic cells that project to peripheral organs, may be involved in the hormonal and metabolic anticipatory changes in preparation for food presentation and thus, may be part of a link between central and peripheral oscillators. In addition, due to their persistent activation they may participate in the neuronal network for the clock mechanism that leads to food entrainment.

## Introduction

Organization of day and night activity is controlled by the suprachiasmatic nucleus (SCN), the master circadian clock located in the anterior hypothalamus. Light is the main environmental zeitgeber that entrains the SCN, which in turn controls behavioral and physiological rhythms in mammals. The SCN have a self-sustaining clockwork mechanism and through humoral and neural projections control local clocks through the body. Thenceforth, the feeding-fasting cycle is temporally organized at the neuroendocrine, physiological and metabolic levels at appropriate times^1^ leading to rhythmic feeding in nocturnal rats which eat at night during their active phase and rest during the day. Likewise, glucocorticoid hormones rise before the onset of activity to provide circulating glucose from liver stores and to promote arousal. During the resting phase circulating glucocorticoid levels drop; glucose is stored as glycogen in the liver and the subjects rest^2^. This orderly sequence controlled by the SCN is challenged by feeding schedules, which are powerful zeitgebers, when the time of food access is restricted to a few hours per day. Therefore, when food is provided during a short period of time during the resting phase, subjects awake and exhibit arousal during the resting phase in anticipation of the time of the scheduled meal in order to obtain food^3^. In addition to intense locomotor behavior, animals show high glucocorticoid circulating levels in advance of meal timing, a phenomenon known as food anticipatory activity (FAA)^4^. As mentioned above, light is the main entraining cue for the master clock. In the case of FAA, food is the main synchronizer, in sharp contrast to the light entrainable system. To date, the neural network underlying the food entrainable oscillator is poorly understood. Moreover, the prevailing evidence better supports the proposal that several oscillators both at the central and the peripheral level are entrained by food and together may drive FAA^5^. The specific network is currently poorly understood as food affects central and peripheral organs, mainly those of the gastrointestinal system and glands that release hormones and metabolites in anticipation and as a response to the restricted meal. This complex array of secretions is one of the reasons that have complicated the identification of specific signals and routes important for a possible locus critical for FAA.

Among the several humoral responses elicited by food ingestion is the activation of hypothalamic cells that produce the hormone oxytocin (OT). It is well documented that food ingestion elicits an acute and sharp activation of OT cells in the hypothalamus and a concomitantly release of this hormone to the peripheral circulation^6,7^. In addition, the infusion of OT exerts anorexigenic effects and their projections from the paraventricular hypothalamic nucleus (PVN) to the brainstem are important for a satiety neural circuit^8,9^. The peripheral actions of OT in relation to food intake are not well explored because attention to this hormone has been centered on its well-known effects in reproductive behavior, particularly upon parturition and lactation. However, in the last few years evidence has accumulated supporting an important role of OT in several metabolic processes (see below); furthermore, here we report that this hormone plays an important and differential process in FAA.

In contrast to the previously well-known activation of OT cells after food intake, recently we reported that a particular subpopulation of oxytocinergic cells in the posterior region of the paraventricular hypothalamus in the rabbit pup shows a clear activation *in anticipation to* their only daily episode of food ingestion^10^. Rabbit pups ingest food only once a day and this has been recognized as a natural model of food entrainment^11,12^. The activation of these OT cells during FAA suggests the presence of a possible oscillatory mechanism in a particular region of the PVN that participates in the preparatory ingestion of the timely upcoming of meal. We suggested that the overall implication of this anticipatory activation of OT cells might be a link between a possible central oscillator and peripheral organs in preparation for metabolic and hormonal events that lead to FAA^10^. We believe that this observation in the rabbit pup is relevant for a better understanding of FAA; however, due to the developmental stage of the rabbit pups, it is not possible to contrast their condition with ad libitum fed and fasted subjects. Likewise, it is not possible to determine the possible persistence of the food entrained patterns as in rodent models. Therefore, we considered it necessary to confirm whether this phenomenon occurs in the rat, which is a species commonly used to study food entrainment.

The present experiment explored OT producing cells in the hypothalamus of adult male rats following a regular restricted scheduled food access protocol to study food entrainment. Based on our previous observations in rabbit pups, our hypothesis was that OT producing cells in the paraventricular hypothalamic nucleus would have a differential activation, as determined by the c-Fos protein, before and after food ingestion in conditions of scheduled food restriction. Moreover, this experimental model allowed us to explore their activation in ad libitum conditions and the persistence of the food entrained pattern after interruption of the scheduled food access.

## Materials and methods

### Subjects and housing

Adult male Wistar rats weighing 275 ± 25 g from the bioterium of the Universidad Veracruzana, were maintained in a controlled light/dark (L/D) cycle (12/12-h, lights on at 07 h, defined as zeitgeber time 0 (ZT0), with regulated temperature (22 ± 1 °C), and with free access to water, and standard rat chow (Rodent Laboratory Chow 5001; Purina, México). Rats were acclimated to environmental conditions for 1 week before starting the experimental procedures, which were approved by the Universidad Veracruzana and conducted according to the National Guide for the Production, Care and Use of laboratory animals (Official Mexican Norm: NOM-062-Z00-1999) and in accordance with the ARRIVE guidelines (https://arriveguidelines.org).

### Groups and food entrainment

**Figure 1:**
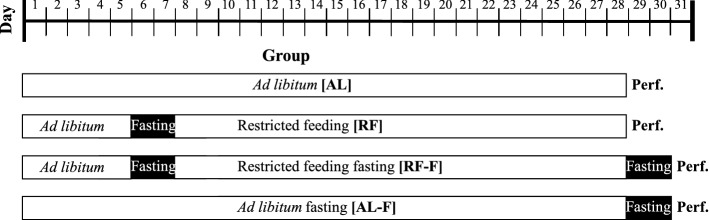
Experimental design. All groups were previously submitted to an adaptation period that consisted of 5 *ad libitum* days followed by 2 fasting days. Restricted feeding groups were fed from ZT5 to ZT7. Perf. = perfusion day.

Rats were randomly assigned to one of four feeding conditions (Fig. 1) and were housed in groups of 4–6 rats of the same condition in transparent acrylic cages (40 × 50 × 20 cm). The ad libitum (AL) group always had free access to food. The food restricted (RF) group had food available daily for 2 h, from ZT5-ZT7, for three weeks. A third group was exposed to the restricted food protocol followed by two days of fasting (RF-F), which was used to determine the possible persistence of c-Fos/OT immunoreactive (-ir) cells, after the termination of the scheduled feeding. Additionally, an ad libitum fasted group (AL-F) was included as a control of the RF-F condition. In this group, after a continuous ad libitum fed condition, food was removed at ZT7, and rats were fasted two days before perfusion. At the end of the manipulation rats were randomly perfused at one of 4 time points (n = 5/time point; a total of 20 subjects /group): ZT1, ZT5, ZT7 and ZT13. The time points ZT1 and ZT5 explored food anticipatory activation, while ZT7 explored the response to food and ZT13 a late response to food intake. General activity was monitored automatically with tilt sensors placed on the ceiling of each individual cage, which detected continuously the movement of subjects. Data were recorded and stored in 15-s bins to generate double plotted actograms and then were grouped in 1 h bins for analysis at the time points indicated with the circadian recording system SPAD9 (Omnialva, México^11^).

### Perfusion and immunohistochemistry

Rats were anaesthetized with an overdose of sodium pentobarbital (Sedal-Vet, 65 mg/ml) and were perfused transcardially with ~ 250 ml of 0.9% saline followed by 250 ml of fixative 4% paraformaldehyde in phosphate buffer (PB, 0.1 M, pH 7.2). Brains were removed immediately after perfusion, postfixed overnight and cryoprotected successively in 10, 20 and 30% sucrose in PB. Brains were frozen at -19 °C and cut coronally at 50 μm thickness with a cryostat (Microm, Walldrof, Germany) from the diagonal band of Broca to the mammillary bodies and one set was processed for immunohistochemistry of c-Fos/OT following protocols previously established for double label immunohistochemistry^13,14^. For c-Fos immunohistochemistry tissue was washed three times for 5 min in PB in order to remove excess aldehydes and then exposed for 10 min in 0.5% hydrogen peroxide solution to eliminate endogenous peroxidase activity. Sections were incubated in the polyclonal c-Fos primary antibody raised in goat at a 1:2000 dilution (sc-52G, Santa Cruz Biotechnology, Santa Cruz, CA, USA) in 3% normal horse serum with 0.3% Triton X-100 (Sigma, St. Louis, MO, USA) at 4 °C. After 72 h tissue was incubated in biotinylated horse anti-goat serum (1:200, Vector Labs, Burlingame, CA) for 1 h and then incubated in the avidin–biotin-HRP complex (1: 250 ABC Vectastain elite, Vector Laboratories) for 2 h. After incubations, tissue was rinsed three times in PB/5 min each. Staining of c-Fos immunoreactivity (c-Fos-ir) was reacted with a solution of 0.05% diaminobenzidine in the presence of nickel sulfate (10 mg/ml, Fisher Scientific, Pittsburgh, PA), cobalt chloride (10 mg/ml, Fisher Scientific) and 0.01% hydrogen peroxide, which produced a black-purple precipitate. After 5 min the tissue was transferred to PB to stop the reaction.

For OT immunohistochemistry sections were rinsed in PB and incubated for 24 h at 4 °C in the monoclonal primary antibody made in mouse diluted at 1:5000 (MAB5296, MERCK) with 3% normal horse serum with 0.3% Triton x-100 (Sigma, St. Louis, MO). Sections were then incubated sequentially in biotinylated secondary anti-mouse serum IgG (1:200, Vector Laboratories) for 1 h, and incubated in the avidin–biotin-HRP complex (1: 250 ABC Vectastain elite, Vector Laboratories, Burlingame, CA) for 2 h. After incubations, tissue was rinsed three times in PB (5 min. each). OT antibody-peroxidase complex reaction was contrasted by using 0.05% diaminobenzidine, which produced a brown cytoplasmic precipitate. Sections were mounted on gelatin-coated slides, dehydrated and cover-slipped with Permount. In all cases, tissue sections from subjects of each ZT time were processed together. As control we used tissue sections processed as above but without primary antibody.

### Quantification of c-Fos, OT and c-Fos/OT cells

Sections were coded, so that the observer was blind to the physiological condition of the subject and were examined under bright-field illumination with an Olympus BX41 microscope at 10X and 20X magnification. c-Fos-ir was identified as a black-purple precipitate from the diaminobenzidine-nickel/cobalt reaction in the cell nucleus, while OT-ir was identified as a brown precipitate in the cytoplasm, whereas double-labeled cells had brown cytoplasm and black nuclei. Immunostaining was absent in tissue processed without primary antibodies. Sections for anatomical identification and quantification of OT-ir, c-Fos-ir and double-labeled c-Fos/OT-ir cells in the hypothalamus were analyzed in the main subdivision of the SON and in two subregions of the PVN, in the median (PVNm) and the posterior region (PVNp). The SON contains magnocellular neurons that project to the posterior pituitary gland. The PVNm contains populations of both magnocellular and parvocellular neurons, and the area was selected on basis of two previous publication from our lab where a strong induction of c-Fos was found after feeding in the rabbit^14^ and in the rat^15^. The PVNp contains preautonomic neurons, which project mainly to the brainstem and spinal cord^16^. The location of brain structures was determined according to the rat atlas of Paxinos and Watson^17^. Sections were analyzed at − 1.40 mm (plate 24), − 1.80 mm (plate 25) and − 2.12 mm (plate 26) for the SON, PVNm and PVNp, respectively. Number of OT-ir, c-Fos-ir and c-Fos/OT-ir cells were counted bilaterally in one representative section at the levels indicated by two observers blind to the experimental condition of animals.

### Data analysis

Cell counts were grouped for each hypothalamic structure, experimental group and time (ZT) and are represented as mean ± standard error of the mean (SEM). Cell count data and locomotor behavior were compared by a two-way-analysis of variance (ANOVA) for factors group X time. The data for number of positive cells at ZT5 or ZT7 were analyzed with-one-way ANOVA. The analysis was followed by a Tukey post hoc test, with significant levels set at P < 0.05. The data that did not pass the homogeneity of variance were rank-transformed before ANOVA analysis^18^. Statistical analyses were performed using Sigma Stat Software version 3.5, (Chicago, IL).

## Results

### Locomotor behavior

**Figure 2:**
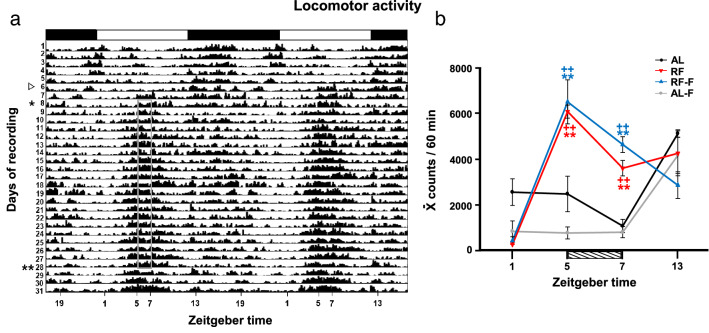
Actogram and locomotor activity. (**a**) Double-plotted actogram showing general locomotion activity of rats under a light/dark cycle with food and water ad libitum during baseline (days 1–5), and then suppressed to food (white arrowhead days 6–7), followed by restricted access to food from ZT5-ZT7 (vertical rectangle), onset indicated by the asterisk (3 weeks), and finally total food deprivation (days 29–30). The horizontal black and white bar above the x-axis denotes the daily LD condition. (**b**) Mean locomotor activity (mean ± SEM for the previous 60 min) for a time point before onset of FAA (ZT1), at the moment (ZT5) and the end (ZT7) of food access and at the onset of the night phase (ZT13). Horizontal stripped bar represents mealtime (from ZT5 to ZT7) for the RF and expected mealtime for the RF-F group. **Indicate significant difference between highest and lowest values for the same group (P < .05). ++Indicate difference between groups at the same time point (P < 0.05).

Rats under AL and AL-F conditions showed an increase of locomotor behavior at the beginning of the night which is the onset of their normal activity phase. In general, it was low during the day with a sharp increase at ZT13 at the onset of the night and remained high along the dark phase. RF and RF-F groups had a completely different pattern. Rats under restricted feeding, RF and in RF-F groups developed FAA at the middle of the day at the time when they are normally sleeping. As shown in the actogram (Fig. 2a) the locomotor behavior started increasing about 2 h before the mealtime (ZT3-4), reaching the maximal activity at the moment of food access. In both groups the highest value at ZT5 (at mealtime for RF or projected mealtime for RF-F), was significantly higher than the time point before (ZT1; P < 0.05) and after feeding (ZT7; P < 0.05). The two-way ANOVA indicated that locomotor activity varied significantly among feeding conditions (F_3-48_ = 11.64, P < 0.001), time factor (F_3-48_ = 28.72, P < 0.001) and the interaction between feeding condition and time (F_3-48_ = 11.52, P < 0.001; (Fig. 2b). The post hoc analysis between groups indicated that values at ZT5 for RF and RF-F groups were significantly higher than AL and AL-F conditions (P < 0.001).

### Supraoptic nucleus (SON)

**Figure 3:**
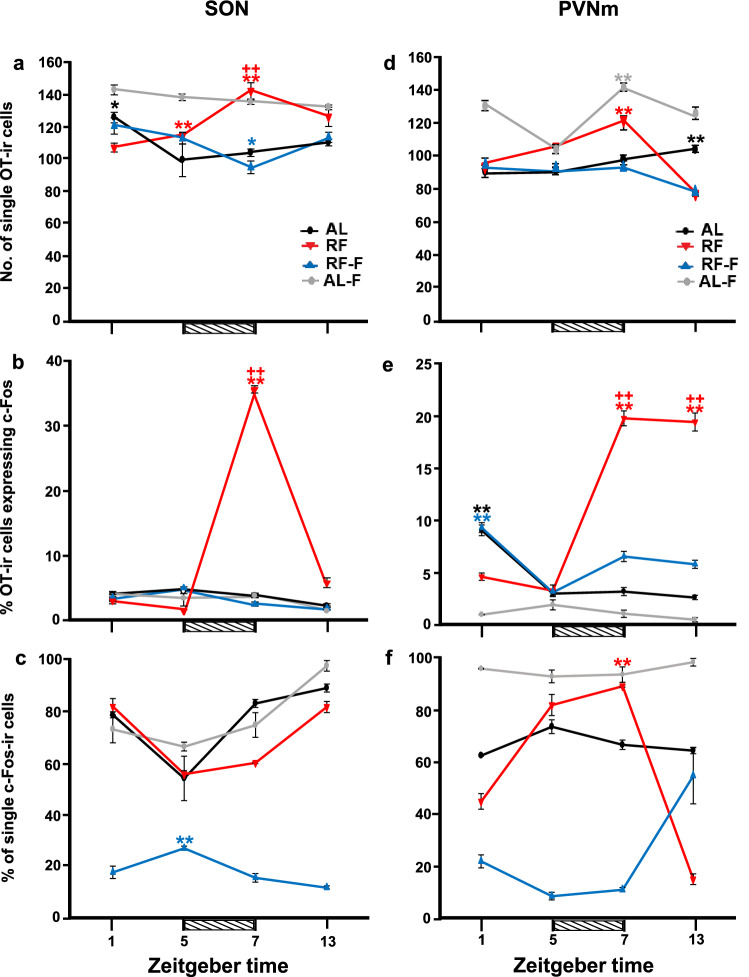
Activation of Supraoptic and Paraventricular hypothalamic nucleus by food intake. Number (mean ± SEM) of single OT (**a**, **d**), percentage of OT expressing c-Fos (**b**, **e**) and percentage of single c-Fos (**c**, **f**) cells in the SON (**a**–**c**) and PVNm (**d**–**f**) in rats kept ad libitum (AL), under restricted food access (RF), under RF followed by 2 days of fasting (RF-F) and ad libitum fed followed by 2 days of fasting (AL-F) at four different zeitgeber times (n = 5 *per* time point). Horizontal stripped bar represents mealtime (from ZT5 to ZT7) for the RF group and expected mealtime for the RF-F group. **Indicate difference between the highest and lowest value within the same group (P < 0.05); ++indicate difference between groups at the same time point (P < 0.05).

In the SON, food intake induced an activation of OT cells after feeding in food-entrained rats. Single OT cell values in the AL group remained constant except at ZT5, which was significantly lower than value at ZT1 (P = 0.02). In the RF group, a significant increase at ZT7 was observed in comparison to values before feeding at ZT1 and at ZT5 (P < 0.01). In the RF-F group the increase observed in RF group at ZT7 did not persist; values were similar in all time points except at ZT7, which was significantly lower than values at ZT1 and at ZT13 (P < 0.05). In the AL-F group, OT values were in general higher than in the other groups. The two-way ANOVA indicated that OT expression in the SON varied significantly due to the group condition (F_3-63_ = 37.98, P < 0.001), and the interaction between feeding condition and time (F_9-63_ = 9.011, P < 0.001), but not by time factor (F_3-63_ = 2.55 P < 0.06; Fig. 3a). Additionally, at ZT7 one-way ANOVA indicated that the number of single OT cells in RF and AL-F groups were significantly higher than those values in the remaining groups (P = 0.001 in both cases; Fig. 7a).

**Figure 4:**
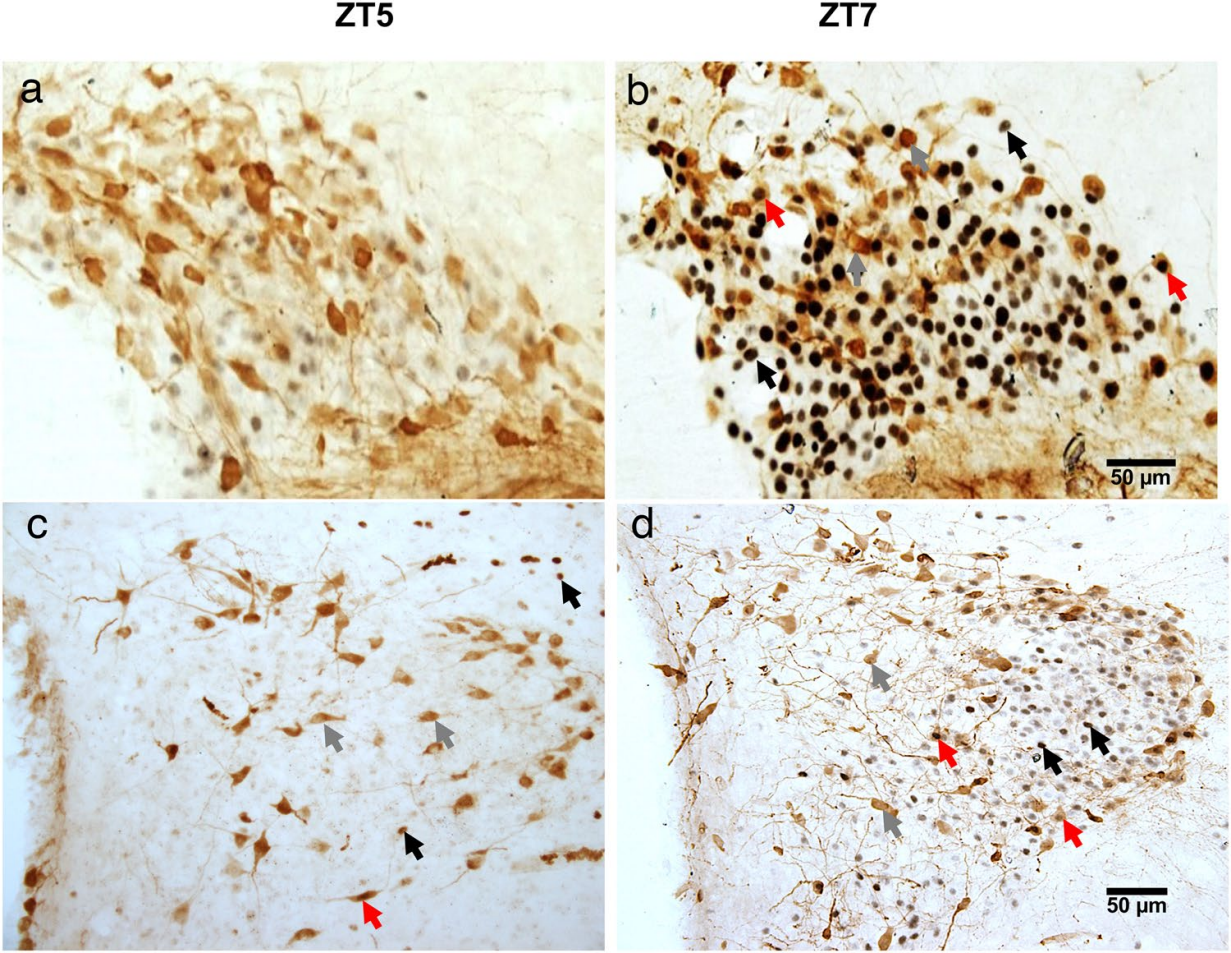
Activation of oxytocin cells by food intake. Representative photomicrographs of SON (**a**,**b**) and PVNm (**c**,**d**), before (**a**,**c**) and after (**b**,**d**) food intake at ZT5 and ZT7, respectively. Note the c-Fos increase in OT cells at ZT7 in both nuclei. Arrows indicate:  = c-Fos,  = OT and  = c-Fos/OT cells.

The percentage of double-labelled c-Fos/OT cells were scarce in all groups except at ZT7 in the RF group where food intake induced a sharp increase (P < 0.001) of double-labelled cells (Fig. 3b). In the RF-F group, the food entrained increase observed in RF group at ZT7 did not persist; the OT % activation was similar than in AL and AL-F groups. The two-way ANOVA indicated that the c-Fos/OT percentage of activation in the SON varied significantly among groups condition (F_3-63_ = 483.75, P < 0.001), time factor (F_3-63_ = 221.37, P < 0.001) and the interaction between groups and time (F_9-63_ = 193.79, P < 0.001; Fig. 3b). In Fig. 4a we show microphotographs of SON at the time of feeding (ZT5) and two hours later at ZT7 (Fig. 4b). Additionally, at ZT7, one-way ANOVA indicated that the number of c-Fos/OT cells in the RF group was significantly higher than that in the remaining groups (P = 0.001; Fig. 7a).

The percentages of single c-Fos cells were high in all groups except in the RF-F group where values were lower, and the value at ZT5 was significantly higher than the remaining time points (P < 0.01). Similar expression patterns were observed in AL, AL-F and RF groups with lower percentages at ZT5 and a steady increase until ZT13. The two-way ANOVA indicated that c-Fos expression in the SON varied significantly with group condition (F_3-63_ = 320.19; P < 0.001), due to the time factor (F_3-63_ = 25.68, P < 0.001) and to the interaction between feeding condition and time (F_9-63_ = 13.00, P < 0.001; Fig. 3c). Additionally, at ZT7 the one-way ANOVA indicated that the number of single c-Fos cells in the RF group, was significantly higher than that in the remaining groups (P < 0.001; Fig. 7a).

### Median subregion of the paraventricular hypothalamic nucleus (PVNm)

Similar as observed in the SON, in the PVNm food intake induced an activation of OT cells after feeding in food-entrained rats. Single OT cell values in the AL group were similar in all time points with only a significant increase at ZT13 in comparison to ZT1 and at ZT5 values (P < 0.001). In the RF group, there was an increase of OT expression at ZT7 that was significantly different from the remaining time points (P < 0.001). In the RF-F group the increase observed in RF group at ZT7 did not persist and values remained unchanged in most time points except at ZT13 which was significantly lower from remaining time points (P < 0.001). In the AL-F group, OT value at ZT7 was significantly higher than remaining time points (P < 0.001 in all cases). The two-way ANOVA indicated that OT expression in the PVNm varied significantly with group condition (F_3-63_ = 235.289, P < 0.001), time factor (F_3-63_ = 56.755, P < 0,001) and the interaction between feeding condition and time (F_9-63_ = 36.760, P < 0.001; Fig. 3d). Additionally, at ZT7 the one-way ANOVA indicated that the number of positive single OT cells in the RF group and AL-F groups were significantly higher than in the remaining groups (P = 0.001 in both cases; Fig. 7b).

Percentage of double-labelled c-Fos/OT in the AL group showed high c-Fos/OT expression at ZT1 that was significantly different from the remaining time point values (P < 0.001; Fig. 3e). In the RF group, food ingestion triggered expression of c-Fos in OT cells at ZT7 and ZT13, which were significantly higher than the values at ZT1 and ZT5 (P < 0.001) and from all groups at ZT7 and ZT13 (P < 0.001). The increase in activation of OT cells at ZT7 did not persist in the RF-F group. In this group the highest OT % activation was observed at ZT1, which was significantly different from the remaining time point values (P < 0.001 in all cases). In the AL-F group, the OT % activation was not significantly different at any time point (P = 0.250). The two-way ANOVA indicated that OT % activation in the PVNm varied significantly with group condition (F_3-63_ = 335.60, P < 0.001), time factor (F_3-63_ = 74.92, P < 0.001) and the interaction between feeding condition and time (F_9-63_ = 116.54, P < 0.001; Fig. 3e). In Fig. 4c,d we show microphotographs of the PVNm at the time of feeding at ZT5 and two hours after at ZT7. Additionally, at ZT7 the one-way ANOVA indicated that the number of c-Fos/OT cells in the RF group was significantly higher from the other groups (P = 0.001 in all cases; Fig. 7b).

The percentage of single c-Fos values showed a different pattern of expression in all groups Fig. 3f). Values were similar in the AL-F group at all time points without significant differences (P > 0.05). In the AL group values were also similar in all time points and the value at ZT5 was significantly higher than values at ZT1 and ZT13 (P < 0.01 in both cases). In contrast, in the RF group there was a sharp increase at ZT5 and ZT7 which was significantly higher than values at ZT1 and ZT13 (P < 0.001 in both cases). In the RF-F group values were low at all time points except at ZT13 (P < 0.001) which was significantly higher than the remaining time points. The two-way ANOVA indicated that in the AL, RF, RF-F and AL-F groups, percentage of single c-Fos in the PVNm varied significantly with group condition (F_3-63_ = 298.64, P < 0.001), time factor (F_3-63_ = 6.68, P < 0.001) and the interaction between feeding condition and time (F_9-63_ = 46.18, P < 0.001; Fig. 3f). Additionally, there was a sharp increase in the number of single c-Fos cells at ZT7 in the RF group which was significantly different than remaining groups (P < 0.001; Fig. 7b).

### Posterior subregion of the paraventricular hypothalamic nucleus (PVNp)

In contrast to the SON and PVNm, the PVNp exhibited a completely different pattern of c-Fos activation in OT cells. In the RF group, there was a sharp induction of c-Fos *before* food intake, at the time of FAA. Importantly, this increase persisted in the RF-F group in fasted conditions at the time of the previous scheduled mealtime.

**Figure 5:**
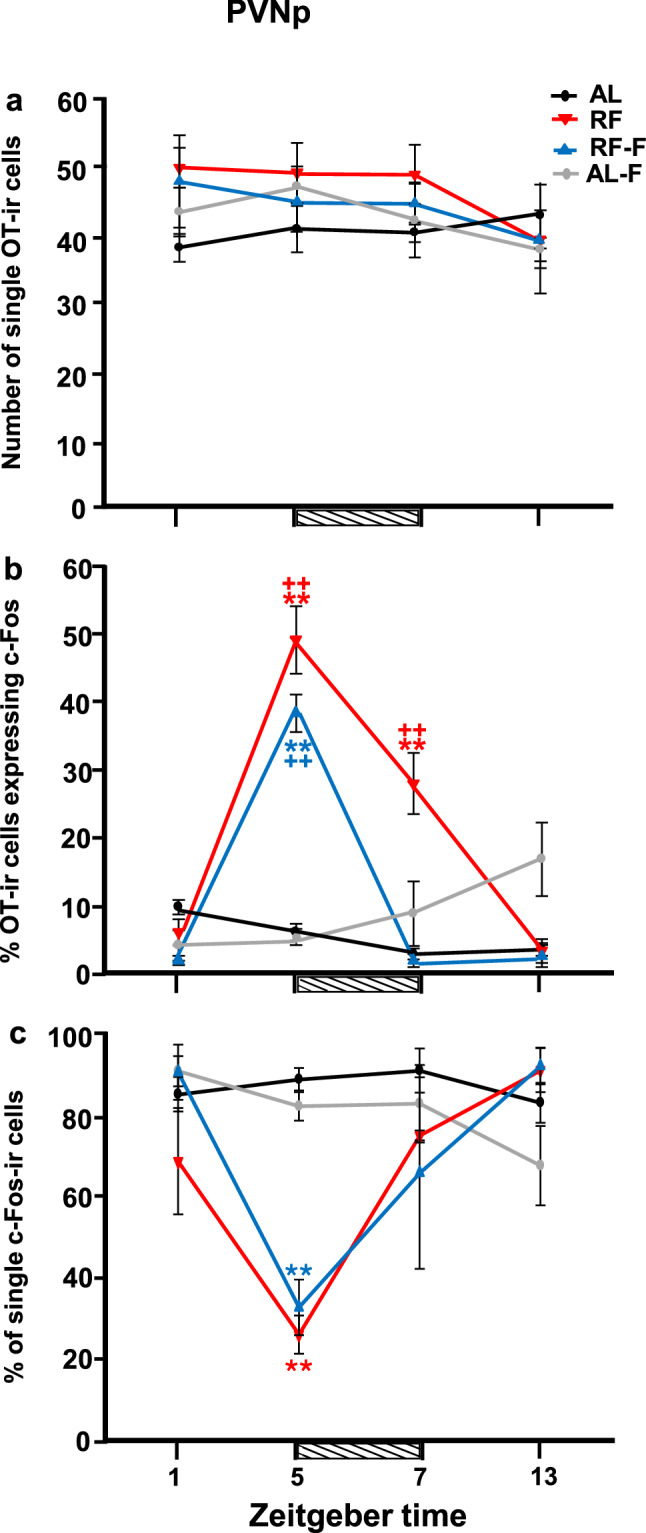
Activation and persistence of OT cells in RF and RF-F subjects in the posterior subregion of the Paraventricular hypothalamic nucleus. Number (mean ± SEM) of single OT (**a**), percentage of OT expressing c-Fos (**b**) and percentage of single c-Fos (**c**) cells in the PVNp in rats kept ad libitum (AL), under restricted food access (RF), under RF followed by 2 days of fasting (RF-F) and ad libitum fed followed by 2 days of fasting (AL-F) at four different zeitgeber times (n = 5 *per* time point). Note the persistence of activation in RF-F rats. Horizontal stripped bar represents mealtime (from ZT5 to ZT7) for the RF group and expected mealtime for the RF-F group. **Indicate difference between the highest and lowest value within the same group (P < 0.05); ++indicate difference between groups at the same time point (P < 0.05).

The number of OT immunoreactive cells was constant across time points for all experimental groups (Fig. 5a). The two-way ANOVA indicated that OT expression in the PVNp did not vary with group condition (F_3-63_ = 1.48, P = 0.23), time factor (F_3-63_ = 1.39, P = 0.25) nor with the interaction between feeding condition and time (F_9-63_ = 0.54, P = 0.83).

**Figure 6:**
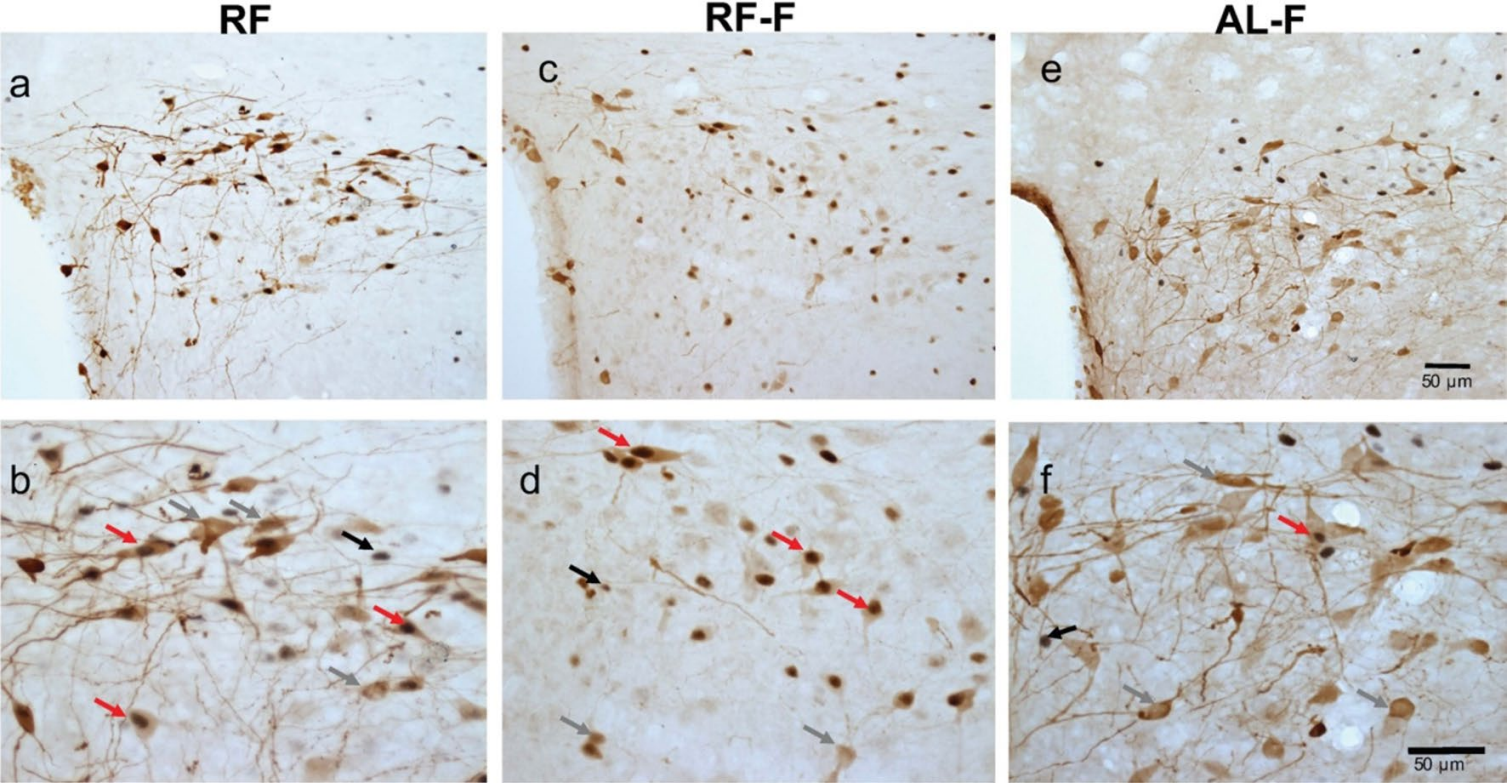
Anticipatory activation of OT cells in RF and their persistence in RF-F subjects in the posterior subregion of the Paraventricular hypothalamic nucleus. Representative photomicrographs of OT, c-Fos and c-Fos/OT cells in PVNp of RF (**a**,**b**), RF-F (**c**,**d**) and AL-F (**e**,**f**) groups at ZT5. Note double-labelled c-Fos/OT cells in RF and RF-F, but not AL-F groups. (**b**,**d**,**f**) Show higher power images of the same photomicrographs in (**a**,**c**,**e**), respectively. Arrows indicate:  = c-Fos,  = OT and  = c-Fos/OT cells.

**Figure 7:**
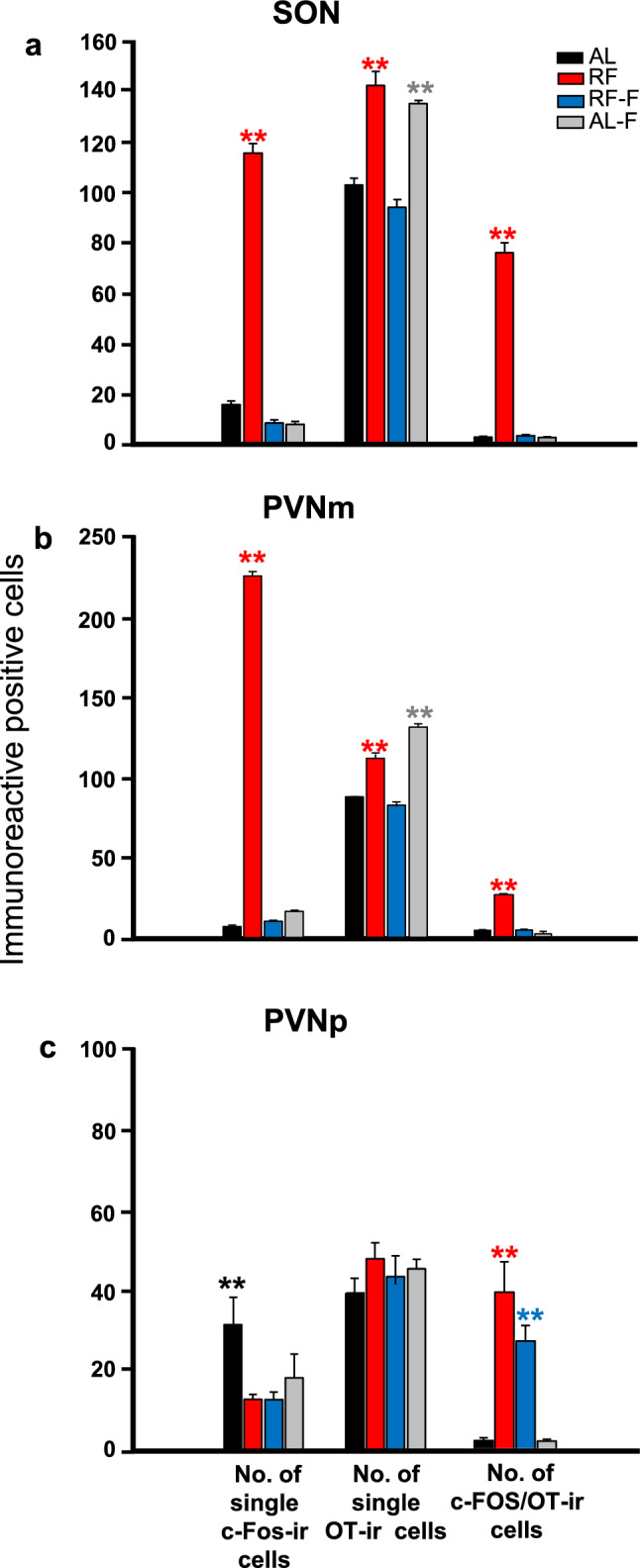
Number of single c-Fos, single OT and c-Fos/OT double labelled cells cells in the SON (**a**), PVNm (**b**) and PVNp (**c**) at ZT7 (**a**,**b**) and ZT5 (**c**) in the AL, RF, RF-F and AL-F groups. Values are mean ± SEM. **Indicate difference between the highest and lowest value within the corresponding cell type (P < 0.05).

In contrast to the SON and PVNm, the PVNp, percentage of c-Fos/OT cells showed an activation *before* feeding at ZT5 in RF group, coinciding with the time of FAA, and this activation persisted in the RF-F group (P < 0.01; Fig. 5b). In addition, food ingestion also showed an increase in activation of OT cells at ZT7 in RF group, which was significantly higher than the values at ZT1 and ZT13 (P < 0.01). This effect was not observed in AL and AL-F groups (Fig. 5b). Double-labelled c-Fos/OT cells in the PVNp at ZT5 are shown in Fig. 6a,b and their persistence in RF-F in Fig. 6c,d. No double-labelled c-Fos/OT cells were observed at ZT5 in the AL-F group (Fig. 6e,f). The two-way ANOVA indicated that c-Fos/OT positive cells in the PVNp varied significantly with group condition (F_3-64_ = 24.60, P < 0.001), time factor (F_3-63_ = 39,18, P < 0.001) and the interaction between feeding condition and time (F_9-63_ = 21.78, P < 0.001). Additionally, at ZT5 the one-way ANOVA indicates that number of c-Fos/OT cells values in RF and RF-F groups were significantly higher than in remaining groups (P = 0.001 in both cases; Fig. 7c).

Percentage of single c-Fos values, were similar in AL and AL-F groups at all time points without significant changes. In contrast in RF and RF-F groups there was a sharp decrease at ZT5 which was significantly different than the remaining time point values (P < 0.05) in RF group and at ZT1 and ZT13 in RF-F group. The two-way ANOVA indicated that the percentage of single c-Fos varied significantly with group condition (F_3-63_ = 5.28, P < 0.01), time factor (F_3-63_ = 8.41, P < 0.001) and the interaction between feeding condition and time (F_9-63_ = 4.62, P < 0.001; Fig. 5c). Additionally, at ZT5 the one-way ANOVA indicates that number of single c-Fos cell value in AL group was significantly higher than in remaining groups (P = 0.001 in all cases; Fig. 7c).

## Discussion

In agreement to our hypothesis, we found a differential activation of OT producing cells in the hypothalamus of adult rats under conditions of food restriction. In the RF group a high proportion of oxytocinergic cells in the SON and in the PVNm expressed c-Fos after food intake. More important, a specific population in the posterior part of the PVN showed c-Fos activation *before* food presentation at the time of FAA. Furthermore, this anticipatory activation persisted in entrained-fasted animals at the previous time of scheduled feeding. Present data indicate a differential role of the medial and posterior regions of the PVN in food entrainment. While the medial region appears to respond to the incoming entraining signal given by food intake, the posterior region was activated in anticipation to the feeding event. Overall, our results contribute to recent accumulating evidence about actions of OT on metabolism homeostasis and are relevant for a better understanding of the FAA phenomenon at both central and peripheral levels.

The activation of OT cells after food intake is in agreement with previous reports showing a strong expression of c-Fos in both the SON^19^ and PVN^15^ in the rat hypothalamus. In the SON and PVN some c-Fos producing neurons were previously identified as oxytocinergic^19^ like present results. Under conditions of food entrainment, a strong induction of c-Fos was detected in the main body of the PVN at a similar level as seen in the present study in the PVNm, but their phenotypical identity was not identified^15^. Here we report that many of those activated cells are oxytocinergic. Classical anatomical studies indicate that OT cells in the SON are magnocellular, project to the neurohypophysis and secrete large amounts of OT, and vasopressin, to the peripheral circulation^16^. But, unlike the SON, the PVN has been shown to be more complex^16^. Here, we observed that OT cells in the PVNp, a region not explored before in food-restricted rats, exhibit a different response from cells in the PVNm.

### SON and PVN have rhythms controlled by the SCN

In AL rats significantly high levels of c-Fos and c-Fos/OT double labelled cells were found at ZT1, during the morning in the SON and PVN, similar to a previous publication in *Ad lib* rats^15^. Both SON and PVN have rhythmic activity controlled by the SCN^1,2^. Anatomical studies show that these two nuclei are targets of the master clock and show parallel c-Fos induction triggered by a light pulse in the SCN^20^. In vitro experiments demonstrate that both SON and PVN show rhythmic activity which rapidly dampens in absence of the SCN input, which confirms that their rhythmic activity is driven by the master clock^21^. Under conditions of food restriction both nuclei uncouple their oscillations from the SCN, although this nucleus still participates in food-entrainment ^22^. In this sense it is interesting that the peptide vasopressin (AVP) innervates OT neurons in the PVN to regulate feeding under normal conditions^23^, a role which needs to be explored in the context of food-entrainment.

### Oxytocin, a key hormone as an entraining cue for food entrainment?

There is a general agreement that food elicited metabolic responses may be the entraining cue in relation to food entrainment^5^. But unlike the light entrainable system, in the case of food the pathways and the entrained targets are broadly distributed at both central and peripheral level, which has complicated understanding of this phenomenon. Also, they have led to the proposal that instead of a single master clock as in the case of the light entrainable system, there are multiple possible food entrained oscillators responding to different humoral and neural pathways^5^. In this regard, it was recognized that very little is known about the synchronizing signals and pathways in food-restricted animals, once food, the main cue, is ingested^5^. Based on our present results, we propose that the release of the hormone OT following food intake could be an important candidate to be considered as one of these synchronizing signals.

Our study shows that under the condition of food restriction, oxytocinergic cells are activated by food intake and shift SON and PVN from their usual daily rhythms controlled by the SCN to a new phase entrained by periodic food intake. Although the SON shows a few c-Fos expressing cells in AL and RF groups at different time points, there is a strong induction of this protein after food intake in agreement to a previous report in *ad libitum*^7^ and food entrained rats^15^. Moreover, previous work from our laboratory revealed that many of those induced cells are oxytocinergic, not only in the SON but also in the PVN in the rabbit pup^14^, as well as in the rat as observed in the present study, suggesting a massive release of OT to the bloodstream^7^.

The attention to the peripheral OT release has been centered in the classical effects of oxytocin on the reproductive system as parturition and milk ejection^24^. Nonetheless, in the last years evidence is accumulating about metabolic effects of this hormone. Circulating OT has shown to improve insulin sensitivity and lipolysis, stimulates glucose uptake and lipid utilization in adipose tissue and skeletal muscle^25^. In addition, the metabolic effects of OT on peripheral organs also can be achieved through indirect projections. The PVN is composed of at least 10 different subregions of magnocellular and parvocellular neurons^26^. In addition to the neurohypophysis, they project to the brainstem and the spinal cord, specifically those in the posterior region of the PVN^16^, and transmit their signal through the autonomic nervous system.

### PVN as a site of preautonomic motor neurons

Electrophysiological studies identified some neurons in the PVNp as preautonomic motor neurons that project to autonomic cells in the brainstem or the spinal cord^27,28^. The attention to these pathways focused mainly on cardiovascular function^29^. Detailed studies by retrograde tracing, electrophysiological recordings and morphological reconstructions characterized the properties of these pre autonomic neurons, which are different from adjacent magnocellular neurosecretory neurons. Moreover, these pre autonomic neurons were identified in the posterior region of the PVN^30^ at similar posterior levels as in the present contribution and in our previous report in the rabbit pup^10^. Further tract tracing studies with selective autonomic denervation identified separate populations of preganglionic sympathetic and parasympathetic motor neurons in the dorsal and posterior PVN, which through the intermediolateral column and the dorsal motor nucleus of the vagus innervate the liver, pancreas and adrenals. Importantly, they were identified as oxytocinergic cells^31,32^. In the rat, daily hypothalamic preautonomic control of plasma glucose concentrations and insulin (rev. in ^33^) has been reported. In agreement, denervation of the sympathetic input to the liver resulted in a disruption of the daily plasma glucose rhythmicity^34^, and it was demonstrated that this rhythmic activity of the PVN is controlled by the SCN^2,33^.

### PVN activation during FAA

Recently^10^ we reported in young rabbits a differential activation of the PVN in relation to restricted food access. The dorsal and the posterior part of the PVN showed an increase of c-Fos in OT neurons at the time of FAA, which persisted in fasted subjects. In contrast, OT cells in the ventral part of the main body of the PVN were activated only after food intake^10^. This differential action of PVN cells in relation to food entrainment had been explored in the rat by *c-Fos* mRNA^35^. These authors found activation of parvocellular cells in the dorsal and caudal portion of the PVN during FAA; in contrast, magnocellular cells in the main body of the PVN, showed activation only after feeding at a similar level here reported. All together, these results reinforce the differential activation of subregions of the PVN at the time of FAA when subjects are hungry and undergoing a catabolic state^36^. Electrophysiological studies have provided evidence about the specific properties of these subregions of the PVN. Patch-clamp recording of PVN neurons in the dorsal posterior portion identified glucosensing preautonomic neurons that are glucose-excited or glucose-inhibited^37^. These glucosensing cells are at a similar level where we reported activation of OT cells during FAA in the rabbit^10^. In sharp contrast, adjacent OT cells of the ventral portion of the PVN, below those activated OT cells, were not activated in the rabbit^10^, nor in the study of Melnick et al.^37^ in rats, which were identified as neurosecretory, not preautonomic and not glucosensitive. Unfortunately, in the work of Melnick et al.^37^ cells of the PVNp were not explored.

Taken together, we propose that OT cells in the PVNp may play a main role during FAA as glucosensitive neurons that may trigger gluconeogenesis through parasympathetic output to the liver. As mentioned during FAA animals are in a catabolic state and exhibit increase of plasma levels of corticosterone, free fatty acids and glucagon whereas there is a decrease in glycogen and insulin^36^ which agree with a possible action of pre autonomic PVN cells on peripheral organs. On the other hand, perhaps, there is an OT release in the brain at ZT5, during FAA, which needs to be explored in future experiments.

### PVN pathway to arcuate nucleus

The injection of the retrograde tracer cholera toxin subunit β into the arcuate nucleus has revealed a novel pathway of OT cells from both the SON and PVN to a large population of proopiomelanocortin (POMC) cells which contains OT receptors^38^. Moreover, the central injection of OT in the lateral ventricle induces c-Fos in POMC and in neuropeptide Y (NPY)/Agouti-related peptide (AGRP) cells. Both POMC and NPY/AgRP are key hormones that regulates food intake and energy expenditure in this feeding center^39^. Future studies are necessary to explore the role of this OT pathway to the arcuate nucleus in the context of FAA.

### Other neurochemicals besides OT

In this study we have reported c-Fos activation in non-oxytocinergic cells in both, the PVNm and PVNp in RF animals. In a preliminary study we found that some of them colocalize with the peptide AVP, without any apparent tendency in the different restricted feeding conditions (data not shown). However, the PVN also contain cells that produce corticotrophin releasing factor (CRF) in addition to other neurotransmitters^26^. Among these cells, CRF neurons are interesting as there is an increase of glucocorticoids during FAA in the rat^40^ and the rabbit^41–43^ and CRF cells are found in the PVN, but not in the SON, and those in the PVN express OT receptors^44^. Additionally, OT but not AVP cells express the corticotrophin releasing factor receptor CRFR2^44^. These authors propose a regulation between OT and CRF release^44^ that may be related to the drop of glucocorticoids in food restricted animals after feeding^36,40,41,43^ or during fasting after a restricted feeding protocol^40,42^ in both the rat^36,40^ and the rabbit ^41–43^. In this regard OT cells are also activated by stressful stimuli^45^; however, as shown in Fig. 3e we observed a significant increase in c-Fos/OT cells in the PVNm only in the RF group after food intake and not by the lack of food in RF-F and AL-F rats.

Moreover, tract-tracing studies with pseudorabies virus in peripheral organs have demonstrated CRF preautonomic cells in the PVN^46^ which also support a possible influence of this nucleus in the increase of glucocorticoids during FAA. Interestingly, the same study^46^ found that in the PVN there are no vasopressinergic preautonomic cells which lead to autonomic innervation of the liver. Overall, this is important evidence that reinforces the importance of OT in the metabolic and hormonal changes during FAA and are also in agreement with the lack of activation of AVP cells in the PVN during FAA, as mentioned above. Interestingly, the injection of 6-Hydroxydopamine in the PVN suppressed the increase of CORT during FAA^47^; this emphasizes the importance of the PVN in FAA, their shift from the SCN influence in animals under food restricted protocols, and also the importance of the catecholaminergic system in the PVN for the hormonal changes during FAA. Future studies need to determine the precise interaction between food intake, OT and CRF cells and their receptors in the PVN. In addition, it is necessary to confirm whether these activated OT cells during FAA and at the time of the previous scheduled feeding time in fasted subjects are indeed pre-autonomic. Lastly, it is necessary to explore the phenotypical identity of c-Fos non-oxytocinergic cells. As shown in Fig. 7 there is c-Fos activation in non-OT cells in the three regions explored, particularly in the PVNm at ZT7. On this regard it is interesting that activation of subsets of PVN neurons expressing nucleobindin2 (NUCB2) and its processing product nesfatin-1 are involved in circadian food intake and regulates OT cells^48^. Moreover, thyrotropin-releasing hormone (TRH) and pituitary adenylate cyclase-activating polypeptide (PACAP) from the PVN, activates NPY/AgRP neurons in the arcuate nucleus regulating feeding in mice^49^. These results agree with the PVN pathway to the arcuate nucleus (see above), that involves other non-OT cells as possible recipients of the entraining signal provided by food intake.

## Concluding remarks

The protocol of food entrainment provided evidence that oxytocin cells in the PVN are a key element in the pathway elicited by food intake, while the PVNm responds to the input provided by meal onset, the PVNp anticipates the scheduled feeding event. In addition, there is activation of non-OT cells, particularly in the PVNm that, besides OT, may play an important role as recipients of the food intake stimulus perhaps through a PVN/Arcuate nucleus pathway. Both regions of the PVN may constitute a possible link between brain and periphery in the coordination with FAA. In addition, cells of the posterior region of the PVN may be a part of the clockwork mechanism for FAA.

## Data Availability

The datasets used during the current study are available from the corresponding author on reasonable request.
